# Elevated Levels of Interferon-γ Production by Memory T Cells Do Not Promote Transplant Tolerance Resistance in Aged Recipients

**DOI:** 10.1371/journal.pone.0082856

**Published:** 2013-12-10

**Authors:** James I. Kim, Ryan T. Stott, Julie Soohoo, Kang Mi Lee, Gaoping Zhao, Heidi Yeh, Shaoping Deng, James F. Markmann

**Affiliations:** 1 Transplantation Unit, Department of Surgery, Massachusetts General Hospital, Harvard Medical School, Boston, Massachusetts, United States of America; 2 Department of Surgery, Sichuan Provincial People’s Hospital and Sichuan Academy of Medical Sciences, Chengdu, Sichuan Province, China; Children's Hospital Boston/Harvard Medical School, United States of America

## Abstract

Immunosenescence predisposes the elderly to infectious and autoimmune diseases and impairs the response to vaccination. We recently demonstrated that ageing also impedes development of transplantation tolerance. Unlike their young counterparts (8-12 weeks of age) aged male recipients (greater than 12 months of age) transplanted with a full MHC-mismatched heart are resistant to tolerance mediated by anti-CD45RB antibody. Surprisingly, either chemical or surgical castration restored tolerance induction to levels observed using young recipients. Based on the strong impact of endocrine modulation on transplant tolerance, we explored the impact of ageing and castration on the immune system. Here we report a significant increase in the percentage of T cells that produce interferon-γ (IFN-γ) in aged male versus young male animals and that the overall increase in IFN-γ production was due to an expansion of IFN-γ-producing memory T cells in aged animals. In contrast to IFN-γ production, we did not observe differences in IL-10 expression in young versus old male mice. We hypothesized that endocrine modulation would diminish the elevated levels of IFN-γ production in aged recipients, however, we observed no significant reduction in the percentage of IFN-γ+ T cells upon castration. Furthermore, we neutralized interferon-γ by antibody and did not observe an effect on graft survival. We conclude that while elevated levels of interferon-γ serves as a marker of tolerance resistance in aged mice, other as yet to be identified factors are responsible for its cause. Defining these factors may be relevant to design of tolerogenic strategies for aged recipients.

## Introduction

The elderly are the fastest growing segment of the population with end-stage organ disorders, and their numbers on the transplant waiting list continue to rise [1–4]. By 2020, for the first time in human history, the number of people older than 65 will outnumber the number of children under 5 [5]. Induction of durable donor specific tolerance could allow successful transplantation without the morbidity of immunosuppression [6,7]. To be broadly applicable, it will need to succeed in recipients of all ages, yet clinical and laboratory transplant tolerance induction protocols almost exclusively rely on young recipients. Furthermore, the majority of basic science research in tolerance takes place in young animals. Thus, in order for tolerance to become a reality for the majority of transplant patients, it is essential to understand the effects of ageing on transplant tolerance. 

Due to a decline in immune function, the elderly are more susceptible to infectious disease and malignancy, while exhibiting an impaired response to vaccination [8–10]. At the cellular level, ageing is associated with a decrease in the number of naive lymphocytes, a decreased proliferation of CD4+CD25- T cells, and a decreased response to mixed lymphocyte reaction [11,12]. This would suggest that tolerance might be more easily achievable in the elderly, but immunosenescence is also accompanied with increased autoimmune disease and cardiovascular disease, in which an over-reactive immune response is thought to play a role perhaps suggesting some loss of regulation [13–15]. In addition, an increase in the ratio of memory to naive T cells (Tnaive) is seen in observed in older humans and mice [16],and memory T cells (Tmem) have a decreased threshold of activation and are resistant to costimulatory blockade [17]. IFN-γ production by memory T cells is also associated with acute renal rejection [18,19]. 

Donor age, recipient age, and donor-recipient age difference all influence graft survival [20–25]. In a study of nearly 49,000 kidney transplant recipients, graft loss associated with acute rejection episodes was considerably higher in elderly recipients; five-year death censored kidney graft survival was 59.9% for recipients over age 65 versus 82% for recipients under age 35 [25]. This suggests that the elderly may require more intense conditioning protocols for tolerance induction, which is complicated by the fact that immunosuppressive therapy also carries higher morbidity for elderly patients [24,26]. 

We have observed that a short course of anti-CD45RB reliably induces stable, robust tolerance to various allogeneic grafts in young mice but never in aged mice [27–29]. In a cardiac transplant model, over half of heart grafts survived long-term in young male recipients, while in old male recipients, all grafts were rejected quickly. Recently, T cell interferon-γ production has been reported to play a key role in age-dependent tolerance resistance [30]. We hypothesized that the increased proportion of IFN-γ producing memory T cells impairs tolerance induction in aged mice, and that castration returns IFN-γ production to normal levels. 

## Materials and Methods

### Mice

Male C3H/HeJ and C57BL/6, aged 8 to 10 weeks, were purchased from Jackson Labs (Bar Harbor, Maine). All mice were housed under specific pathogen-free conditions in approved plastic cages in the animal facility of Massachusetts General Hospital. Mice were randomized to control and experimental groups. Mice were housed 4 per cage under standard conditions at constant temperature, humidity, and light/dark cycles, and provided with food and water *ad libitum*. 

All protocols detailed below were performed following the principles of laboratory animal care and approved by the MGH Institutional Animal Care and Use Committee (#2007N000023). The protocol conforms to the USDA Animal Welfare Act, PHS Policy on Humane Care and Use of Laboratory Animals, the “ILAR Guide for the Care and Use of Laboratory Animals” and other applicable laws and regulations. All efforts were made to minimize suffering. 

### Transplantation

Skin grafts were transplanted to mice according to the technique of Billingham and Medawar [31] as previously described. 100 ug of anti-CD45RB (HB220) and 250 ug of anti-CD40L (MR1) was administered on days 0, 1, 3, 5, and 7 post-transplant; both antibodies were from BioXCell (Lebanon, NH). Anti-interferon-γ (XMG1.2, BioXCell) was administered on days 0, 1, 3, 5, and 7 post-transplant either at 200 ug or 600 ug per dose. Euthanasia was by cervical dislocation under Avertin anesthesia (125-250 mg/kg IP). 

For pain management, animals designated for transplant were administered pre-emptive analgesia for post-operative pain control. The first dose of analgesic was given 30 minutes prior to the initiation of the surgical procedure. Buprenex 0.05 to 0.1 mg/kg sc q 8-12 hrs was administered for the first 3 days post-procedure. In addition, the animals were kept warm and monitored for one hour post-transplant, then at least daily for the first week to assess the general health status of the mouse as well as look for signs of distress. After the first week, the animals were examined 2-3 times per week until graft rejection. 

A skin graft is not a life-threatening procedure, and transplant recipients recover quickly and generally do not die. Any animal in the study that exhibited lethargy, weight loss, ruffled fur, an abnormal appearance, or any other serious health condition was euthanized humanely. 

### Cell preparation and flow cytometry

Single cell suspensions were generated from spleens and peripheral lymph nodes by passage through a 70μM nylon cell strainer followed by RBC lysis buffer (Sigma-Aldrich). Cells were stimulated in Complete Medium (RPMI 1640 containing 10% fetal bovine serum (HyClone FetalClone III, Thermo Scientific), 50 μM 2-mercaptoethanol (ACROS Organics), 1 mM sodium pyruvate, NEAA, 2 mM L-glutamine, 100 IU mL^-1^ Penicillin, and 100 μg mL^-1^ Streptomycin, all from MP Biomedicals) with PMA (50 ng mL^-1^, Sigma), ionomycin (1 μg mL^-1^, Sigma), monensin (GolgiStop; 4 μg mL^-1^, BD), and either with or without LPS (10 μg mL^-1^ *Escherichia coli* serotype 0111: B4, Sigma) in 6 well tissue culture treated plates for 5 hours in a 37° C / 5% CO_2_ incubator. 

Cells were surface stained at 4°C in FACS buffer (PBS containing 2% FBS and 0.1% sodium azide) in 96 well plates, with 2 x 10^6^ cells per well. Fc receptors were blocked with purified anti-Mouse CD16/CD32 (2.4G2, BD Pharmingen) prior to cell surface staining. Fluorescently labeled mAbs were used for the following markers: CD4 (GK1.5, Biolegend), B220 (RA3-6B2, Biolegend), CD8 (53-6.7, eBioscience), CD62L (MEL-14, eBioscience), and CD44 (IM7, BD). 

Intracellular staining was performed using the Intracellular Staining Buffer Set (eBioscience) according to the manufacturer’s instructions. Cells were stained with fluorescently labeled mAbs or non-specific isotypes for the following markers: IFN-γ (XMG1.2), IL-10 (JES5-16E3), all from eBioscience. Samples were run on a LSRII flow cytometer (BD Biosciences) and analyzed using FlowJo software (Tree Star, Inc.).

### Statistical analysis

Data were analyzed using GraphPad Prism (version 5, GraphPad Software). Graft survival between experimental groups was compared using Kaplan-Meier survival curves and Wilcoxon statistics. Other differences between experimental groups were analyzed using the Student’s *t* test. *P* values less than 0.05 were considered statistically significant.

## Results

### Ageing impairs tolerance induction to skin grafts

**Figure 1:**
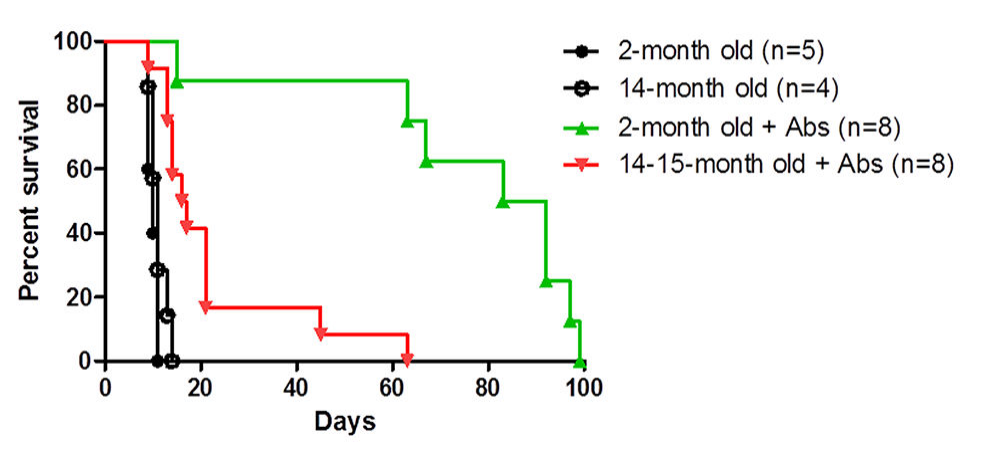
In contrast to young recipients, aged recipients are resistant to transplant tolerance induction. Young C57BL/6 recipients (less than 3-months of age) and aged C57BL/6 recipients (over 12-months of age) reject C3H/HeJ skin graft at same tempo. When treated with anti-CD45RB and anti-CD154 antibodies, 50% of skin grafts survive over 80 days. In contrast, antibody-treated aged recipients reject quickly (aged MST = 16.5 days versus young MST = 87.5 days, p>0.001**).

As previously reported, aged recipients are resistant to anti-CD45RB antibody-mediated transplant tolerance to heart allografts. In young anti-CD45RB-treated C57BL/6 recipients, roughly 60% of C3H/H3J heart grafts survive long-term (>100 days), while C3H/HeJ heart grafts are rapidly rejected in aged recipients (greater than one year-old [29]) receiving the same antibody treatment. Using the same strain combination, we examined whether age-dependent tolerance resistance could also be extended to skin grafts. Both untreated young and old C57BL/6 skin graft recipients reject C3H/HeJ skin grafts at the same tempo (Figure **1**). C3H/HeJ donors are less than three months old. When treated with anti-CD45RB plus anti-CD40L, 50% of young C57BL/6 recipients grafted with C3H/HeJ skin survive greater than 80 days, however, median survival time for antibody-treated aged recipients is 16.5 days (Figure 1). Neither antibody extends graft survival in young recipients (data not shown). Thus, as with heart grafts, aged recipients are also resistant to prolongation of skin graft survival. 

### Elevated production of IFN-γ in aged animals

**Figure 2:**
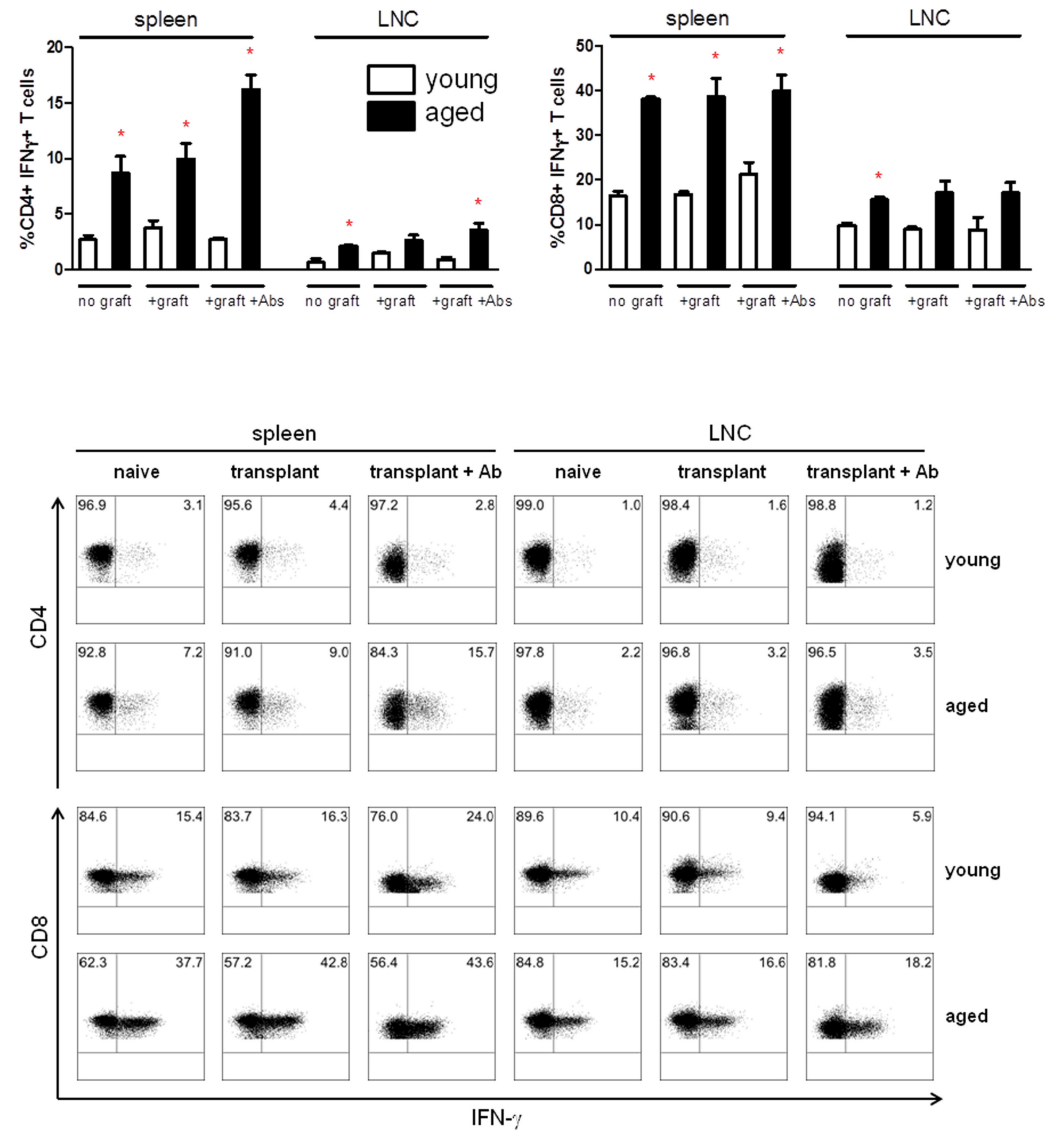
A higher percentage of both CD4 and CD8 T cells from aged animals produce IFN-γ relative to those from young animals. Spleen and lymph node (LNC) from naive animals, grafted animals, and grafted, anti-CD45RB / anti-CD154-treated animals were examined 14 days after transplant, p<0.05*. Representative FACS plots are shown, *bottom*. 2 to 3 animals were examined independently in each group.

To understand the basis for this resistance to tolerance induction in old mice, we examined whether their T cell cytokine profile differed from that of T cells from young mice. We compared interferon-γ production by CD4+ and CD8+ T cells in spleen and lymph node (LNC) of young versus old mice. IFN-γ production by memory T cells has correlated with acute renal rejection [18,19], and we hypothesized that IFN-γ production in aged animals would be higher than in young. Overall, the percentages of lymphocytes comprising the spleen were not significantly altered, but we did observe a decrease in the percentage of CD4+ T cells in aged naive male mice (Figure S1). In naive male animals, a significantly higher percentage of CD4+ and CD8+ T cells from aged mice produced IFN-γ than from young mice (Figure **2**) suggesting a possible mechanism of tolerance resistance in aged recipients. 

### Memory T cell expansion results in elevated IFN-γ production by aged animals

**Figure 3:**
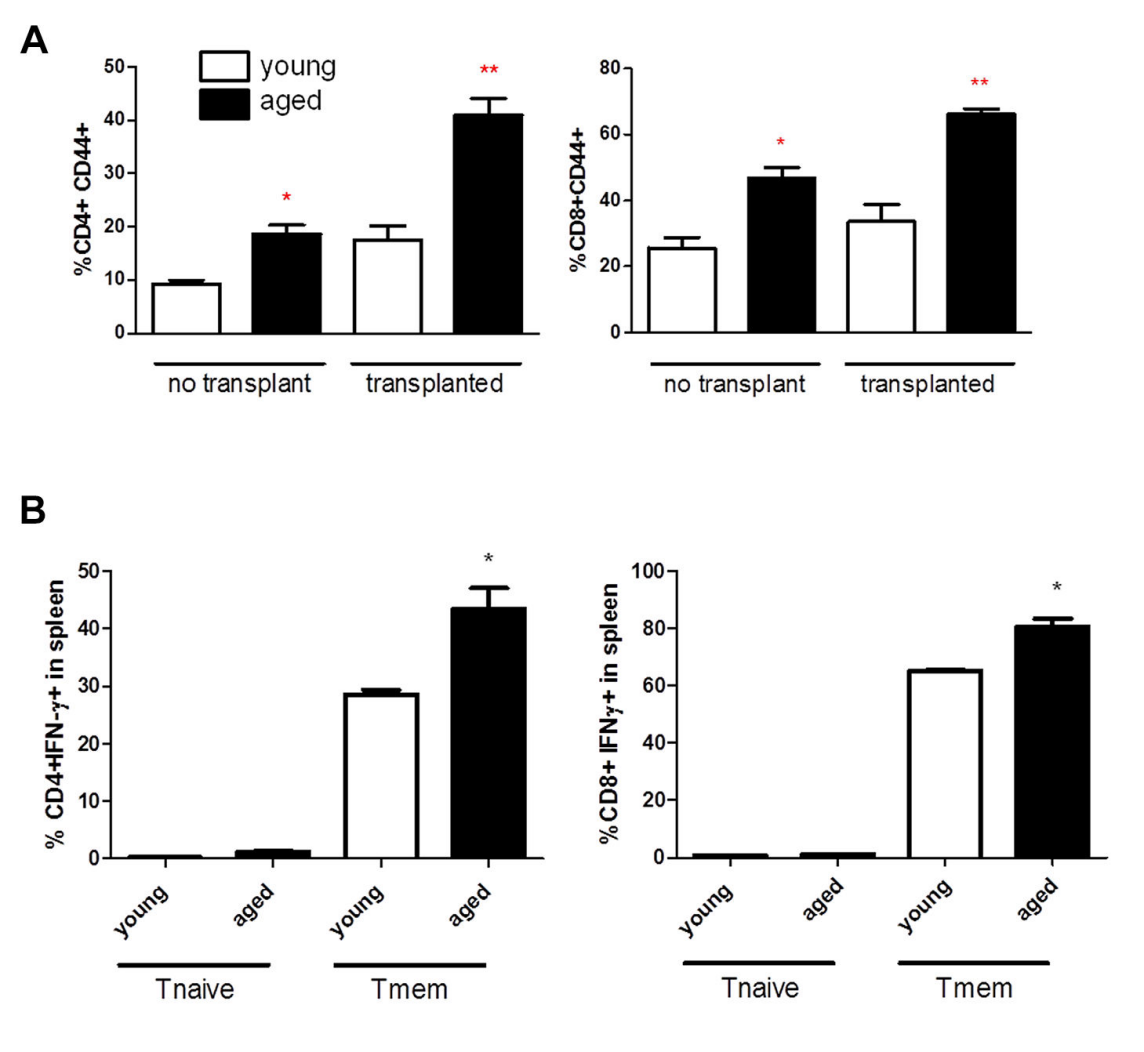
Memory T, not naive T, exhibit elevated production of IFN-γ, in young and old animals. (**A**) CD4 and CD8 memory T cells expand in aged animals. Young and aged animals, with and without skin graft, were examined for memory T cells 14 days post-transplant. At least two to three animals were examined independently per group. (**B**) Splenocytes were examined for CD4, CD8, CD44, and IFN-γ expression. Tnaive cells were gated as CD4+ CD44low or CD8+ CD44low, while Tmem were gated as CD44hi. The percentage of IFN-γ+ cells is plotted on the y-axis. At least two to three animals were examined independently per group.

We next examined which subset of T cells - Tnaive or Tmem - produced the elevated levels of IFN-γ. Tmem have a decreased threshold of activation and are resistant to costimulatory blockade [17], thus, an increased percentage of Tmem would play a significant contribution to transplant tolerance resistance. Spleens from naive aged mice, despite being housed in germ-free conditions, exhibited a significant increase in the percentage of both CD4+ and CD8+ memory T cells (Figure **3A**) compared to naive young mice. Lymph nodes were not examined.

We next examined whether the frequency of IFN-γ-producing Tmem or Tnaive was higher in aged animals versus naive animals. By gating on naive CD4+ and CD8+ T cells in unchallenged young and old animals we found a higher percentage of naive T cells produce IFN-γ in aged animals compared to young animals, yet overall fewer than 2% of the naive T cell populations produced IFN-γ (Figure **3B**). When we examined IFN-γ production by memory T cells, we observed a significantly higher percentage of IFN-γ+ memory CD4+ and CD8+ T cells in aged mice compared to those in young mice. These data suggest that IFN-γ production by Tmem may be involved in age-dependent transplant tolerance resistance. 

Next we examined the impact of transplantation on the percentage of Tmem. We hypothesized that memory T cells in transplanted aged recipients would exhibit more robust expansion than memory T cells in young recipients. Male mice received a skin graft, and spleens were analyzed at two weeks post-transplantation. In young recipients, there is an increase in both CD4+ and CD8+ Tmem after skin graft transplantation which was not statistically significant. In contrast, in aged recipients, there is a statistically significant increase in both CD4+ and CD8+ Tmem after transplantation (Figure **3A**). Most importantly, IFN-γ production by CD4+ T cells of transplanted aged recipients rises with antibody treatment, while young recipients' CD4+IFN-γ production decreases back to baseline levels (Figure 2). These data suggest that the increase in tolerance resistance in aged mice may be related to the failure of antibody treatment to control IFN-γ production by Tmem. 

**Figure 4:**
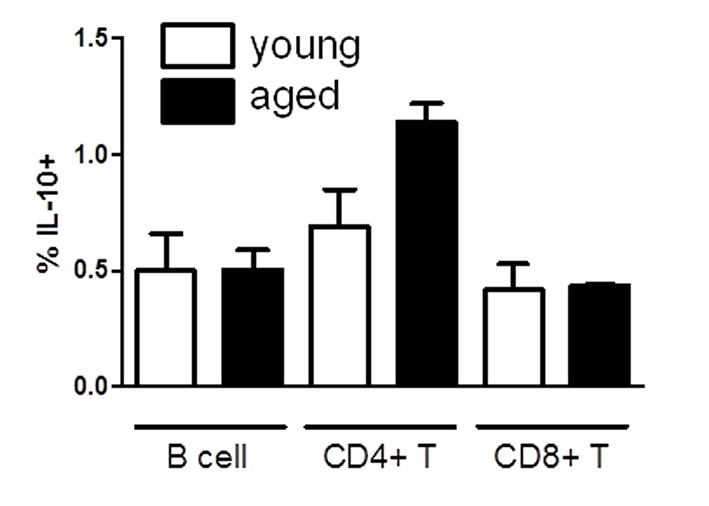
Young and aged animals exhibit similar levels of IL-10 production. Spleen was examined for B220, CD4, CD8, and IL-10. Y-axis indicates percentage of B220+, CD4+, or CD8+ cells that were IL-10+. At least two to three animals were examined independently per group.

We next examined whether IL-10 levels were altered in young male mice versus aged male mice. IL-10 is also a critical cytokine in Th1/Th2 development, and elevated levels of IL-10 and Th2 cytokines correlates with prolonged graft survival [32–34]. We hypothesized that IL-10 production would be significantly lower in aged mice as a result of elevated IFN-γ production. IL-10 levels by B cells and CD4+ and CD8+ T cells were unchanged in young male versus aged male mice (Figure **4**). 

### Castration does not significantly modulate percentage of IFN-γ-producing T cells

**Figure 5:**
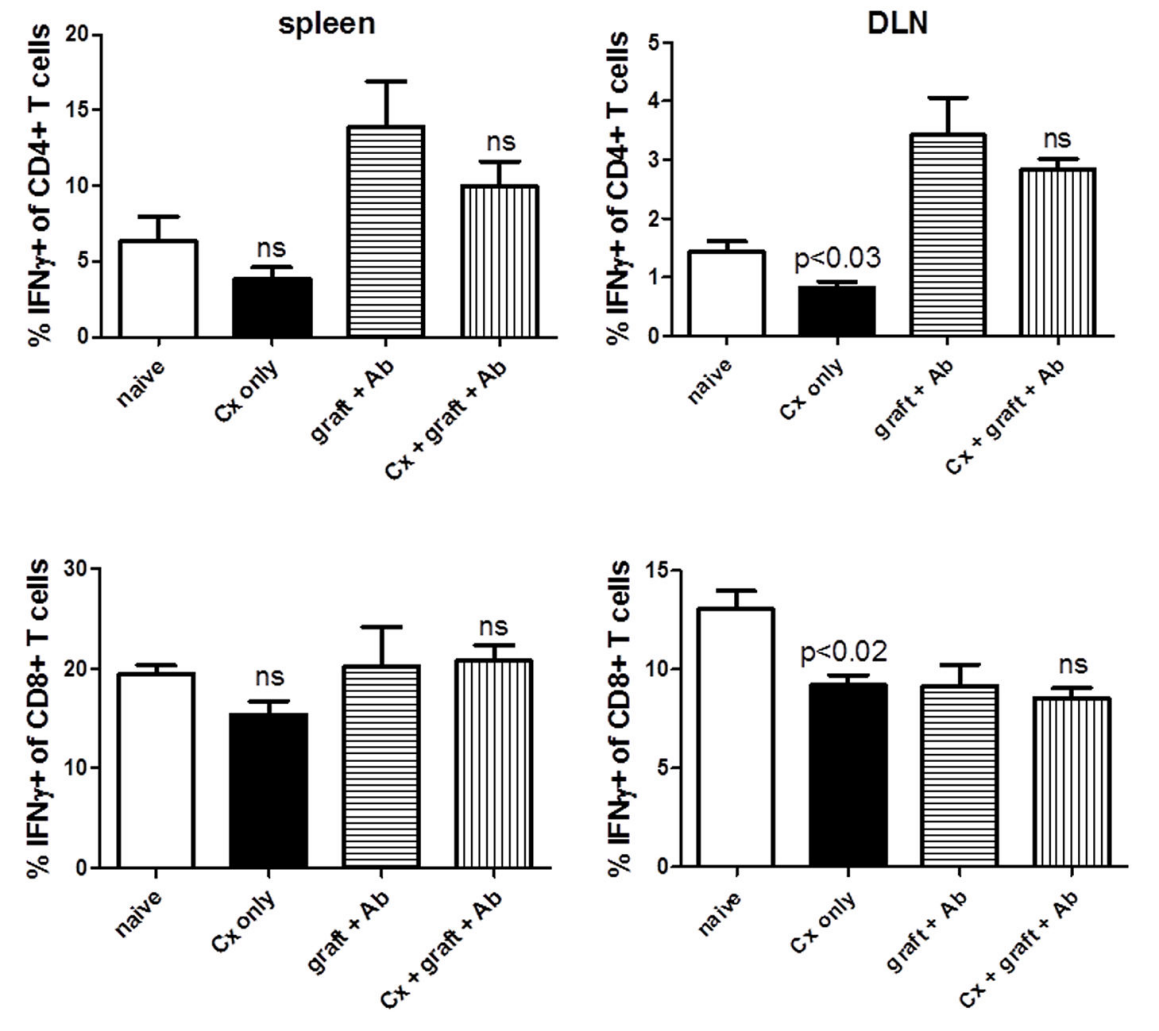
Castration does not significantly modulate levels of CD4+ IFN-γ+ and CD8+ IFN-γ+ T cells. The effect of castration was examined on CD4+ and CD8+ T cells from spleen and lymph node of ungrafted and grafted, antibody-treated animals. Intracellular IFN-γ was examined two weeks after skin graft. Castration was performed 30 days before skin graft. In the absence of a skin graft, castration reduced the % of IFN-γ+ T cells in the lymph node, however, overall, castration did not significantly affect the percentages of IFN-γ production.

We have previously demonstrated that castration restores tolerance to aged, tolerance-resistant recipients [29]. We examined whether modulation of IFN-γ production correlated with the restoration of tolerance. Grafted and non-grafted aged animals were castrated, and spleen and draining lymph node were examined two weeks post-transplant. Castration was performed 30 days prior to transplant. Grafted C57BL/6 mice were transplanted with C3H skin grafts and treated with anti-CD40L / anti-CD45RB antibodies for one week. In the absence of a skin graft, castration did reduce the percentage of IFN-γ+ T cells in the draining lymph node, however, overall, and especially in the transplant setting, there was no significant difference in castrated versus non-castrated groups (Figure **5**). When we specifically examining activated CD4+ CD44+ T cells, still we did not observe any significant difference between castrated and non-castrated groups (data not shown). Thus, these data suggest the elevated levels of IFN-γ-producing T cells may not be the cause of tolerance resistance in aged transplant recipients. 

### Neutralization of IFN-γ does not restore antibody-mediated tolerance induction

**Figure 6:**
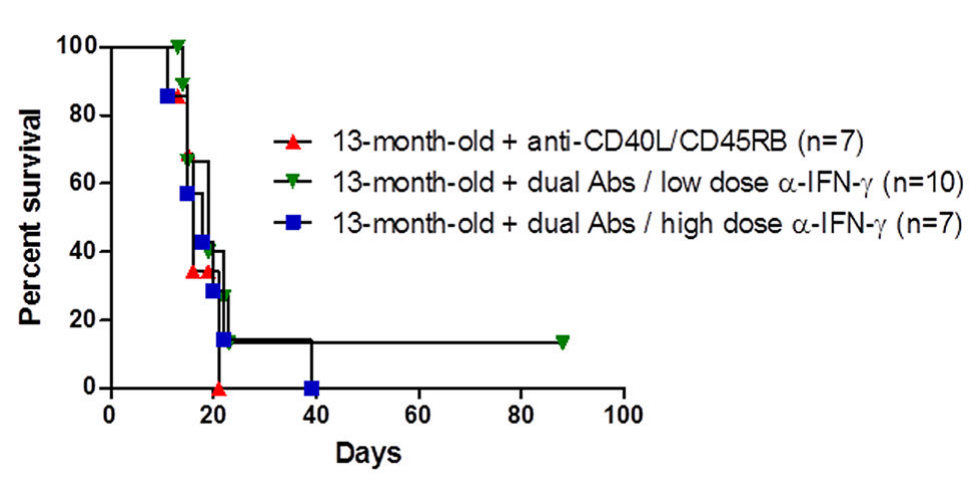
Neutralizing anti-interferon-γ antibody does not prolong graft survival in aged recipients. C57BL/6 mice received C3H skin grafts and were treated with anti-CD40L/anti-CD45RB antibodies with or without anti-interferon-γ antibodies (low dose at 200 ug or high dose at 600 ug). All antibodies were injected every other day for one week starting on the day of transplant.

To further define the role of increased IFN-γ secretion, we neutralized this cytokine in the aged animals by anti-interferon-γ neutralizing antibody. We used two different doses of anti-IFN-γ antibody (clone XMG1.2) to see if we could prolong graft survival as well as to see if there was a dosage effect. We utilized a previously reported regimen [35], and the high dose of anti-IFN-γ antibody was sufficient to accelerate graft rejection in a young recipient (Figure S2). C57BL/6 mice were transplanted with C3H skin grafts and treated with anti-CD40L / anti-CD45RB antibodies with or without co-injection of anti-interferon-γ neutralizing antibody (Figure **6**). While we observed a modest prolongation in median survival time, neither dose of anti-interferon-γ resulted in a statistically significant prolongation of skin graft survival. 

## Discussion

Our work and the work of others suggest that anti-CD45RB treatment promotes the differentiation of Th2 cells and that this Th1/Th2 shift is critical to the tolerogenic effect of anti-CD45RB Ab [36]. Both in human and in mouse, ageing has been demonstrated to be associated with a Th1-Th2 imbalance or a diminished Th1-Th2 response [37], but the data are conflicting. Aged humans have been reported to have increased Th1 [37,38], decreased Th1 [39], increased Th2 [37,38], and decreased Th2 [39]. Similar conflicting reports exist for mice, but such tendencies also result from mouse strain and experimental model differences [40–44]. The effects of ageing on Th1/Th2 in an allograft response remain unclear. Our data suggest that anti-CD45RB antibody maintains low levels of CD4 IFN-γ, a hallmark Th1 cytokine, in grafted young recipients (Figure 2). However, upon antibody treatment, aged, grafted animals produce significantly elevated levels of IFN-γ relative to young, grafted, antibody treated animals (**p<0.01) [19,30]. The molecular mechanism underlying this elevated IFN-γ expression remains unknown. 

Interferon-γ is a potent inducer of cellular immunity. IFN-γ may act directly to promote CD8 T cell responses, and it may act indirectly by skewing CD4 Th cells towards Th1 [45]. In young recipients treated with anti-IFN-γ neutralizing antibody or in IFN-γ-deficient recipients, T cell costimulatory blockade resulted in decreased survival of allografts relative to wild-type or untreated recipients. It was hypothesized that the absence of interferon-γ resulted in uncontrolled proliferation of activated T cells and subsequent accelerated rejection [46–48]. Interferon-γ may also act on the graft to promote its survival [49]. Consistent with this, we found that treatment of young C57BL/6 recipients with high dose anti-interferon-γ resulted in more rapid rejection. 

Memory CD8 T cells have been demonstrated to secrete high levels of IL-2 and IFN-γ in both mouse and in human [8,50,51]. Our *in vitro* data demonstrate that CD4 and CD8 Tmem, particularly Tmem of aged animals, are skewed towards the production of IFN-γ. While both Tmem and Tnaive may differentiate into IFN-γ-producing cells *in vivo*, because Tmem respond more quickly, they are likely to skew the overall response. However, this high proportion of IFN-γ-producing T cells may be due to the genetic propensity of B6 to skew towards Th1. In humans, the frequency of pre-transplant Tmem, as measure by *in vitro* IFN-γ production, correlated with risk of acute renal allograft rejection [18,19]. 

Despite housing in a germ-free facility, Tmem in aged animals are likely to arise either through heterologous activation to an infectious agent or by bystander proliferation [52–55]. Yet since these Tmem were not generated by sensitization to a previous transplant, it is surprising that these cells would still pose such a formidable barrier to transplant tolerance [56]. The barrier is unlikely due simply to precursor frequency of alloreactive cells. By ELISpot, Du et al. demonstrated that the frequency of alloreactive cells in aged mice pre-transplant was no greater than that in young animals. Consistent with survival data, only after transplant and Ab treatment was there a significant difference in the frequency of alloreactive cells between young and aged animals. 

We hypothesized that the increased IFN-γ production that we observed in aged mice was responsible for tolerance resistance, and that the IFN-γ production might in turn be controlled by hormones. However, neither dose of anti-IFN-γ prolonged survival in aged recipients (Figure 6). Most skin grafts survived beyond 15 days, and potentially a dosing regimen in which anti-IFN-γ antibody was administered beyond just the first week might have significantly prolonged graft survival. Castration also had no effect on IFN-γ production in aged mice, further suggesting IFN-γ alone does not mediate tolerance resistance in aged mice, since castrated, aged mice are no longer resistant to tolerance induction. 

Based on our data and the work of others in the effects of ageing on allograft transplantation outcome, we hypothesized that elevated T cell interferon-γ production interfered with tolerance induction. However, our data suggest that this elevated IFN-γ production may not contribute to the tolerance resistance observed with age, and perhaps interferon-γ is but a marker of memory T cells which accumulate with age. Indeed, IFN-γ neutralization did prolong graft survival in aged mice. More potent therapies involving T cell depletion and costimulatory blockade of ICOS and CD28/B7 have demonstrated significant graft survival prolongation in young recipients and should be examined in old recipients [57,58]. 

At the time of puberty and linked with an increase in sex steroid levels, the thymus atrophies [59,60]. This dramatic age-related change results in a significant reduction in thymic size, overall thymocyte cell number, and the absolute number of single-positive CD4+ and CD8+ T cells that leave the thymus [61,62]. Castration has been demonstrated to restore thymic architecture, improve adaptive immunity in both sexes, alter lymphocyte cytokine profile, and in some cases, reverse the Th1/Th2 differences [49,60,63–66]. We previously reported that castration did restore thymic architecture. Our current finding that neutralization of IFN-γ does not affect tolerance resistance and that castration does not change IFN-γ levels would tend to favor the importance of thymic rejuvenation and output in the immunological alterations seen in aged mice.

## Supporting Information

Figure S1 **The percentages of lymphocytes in the spleen of an old mouse are not significantly different from that of a young mouse.** Antibodies to CD4, CD8, and B220 were used to stain splenocytes of young and old mice, and cells were analyzed by flow cytometry. Percentage of CD4+ cells decreased with age (20.7%+/-1.1 versus 14.5%+/-0.9, p<0.01). Data represent two independent experiments and 5 mice. Young mice were 2 months of age, and old mice were over 12 months of age.
(TIF)

Figure S2 **High dose anti-IFN-gamma accelerates graft rejection.** Young C57BL/6 mice were grafted with C3H/HeJ skin and treated with anti-CD40L plus anti-CD45RB antibodies with or without anti-IFN-gamma antibody. Recipients receiving additional IFN-gamma antibody exhibited accelerated graft rejection.
(TIF)
