# Supplementary figures and images for: Elevated Levels of Interferon-γ Production by Memory T Cells Do Not Promote Transplant Tolerance Resistance in Aged Recipients
Source: PLoS One. 2013 Dec 10;8(12):e82856. doi: 10.1371/journal.pone.0082856 (PMC3858330; doi:10.1371/journal.pone.0082856)

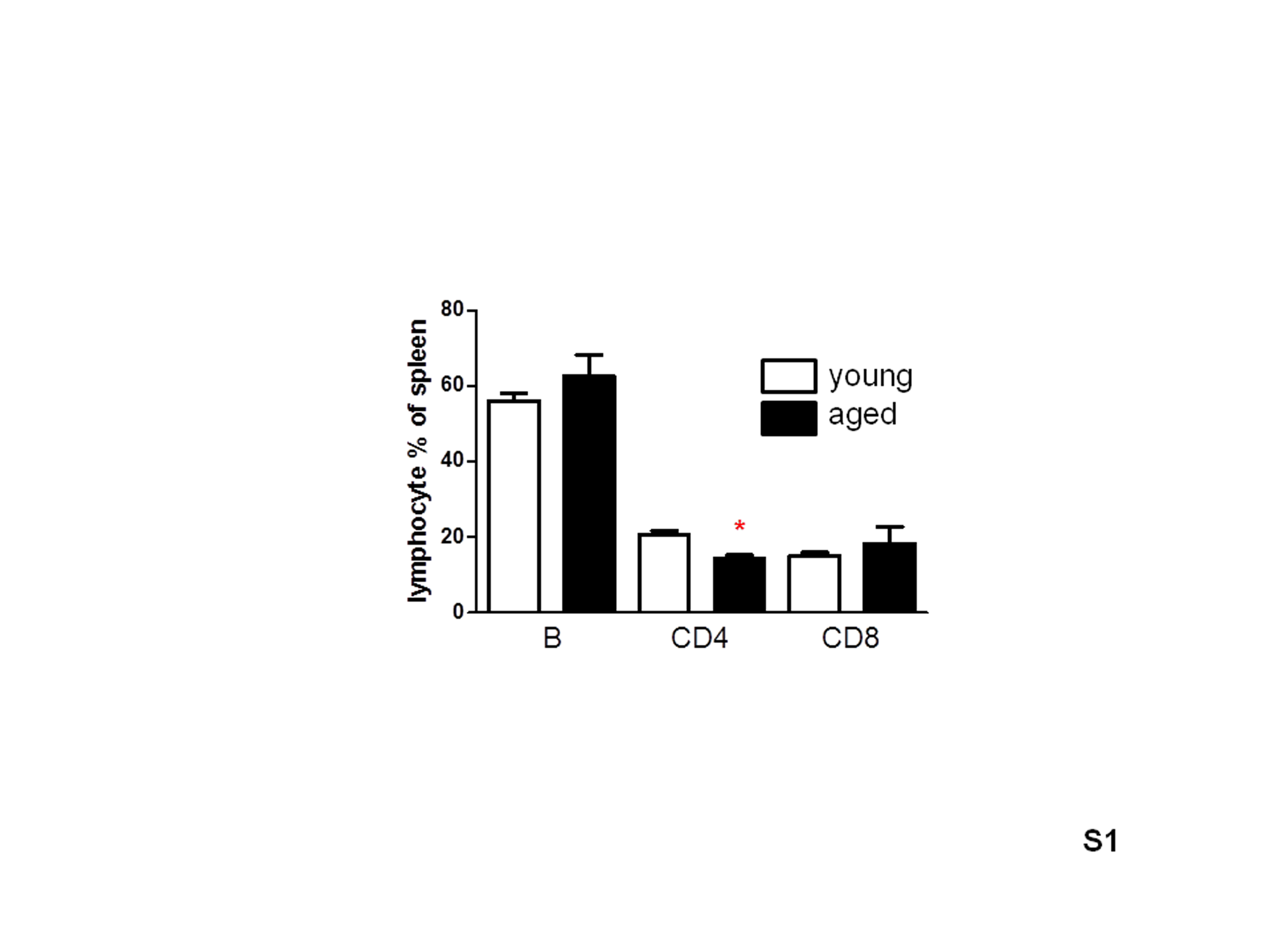

Supplement: Figure S1 — The percentages of lymphocytes in the spleen of an old mouse are not significantly different from that of a young mouse. Antibodies to CD4, CD8, and B220 were used to stain splenocytes of young and old mice, and cells were analyzed by flow cytometry. Percentage of CD4+ cells decreased with age (20.7%+/-1.1 versus 14.5%+/-0.9, p<0.01). Data represent two independent experiments and 5 mice. Young mice were 2 months of age, and old mice were over 12 months of age. (TIF) [file pone.0082856.s001.tif]

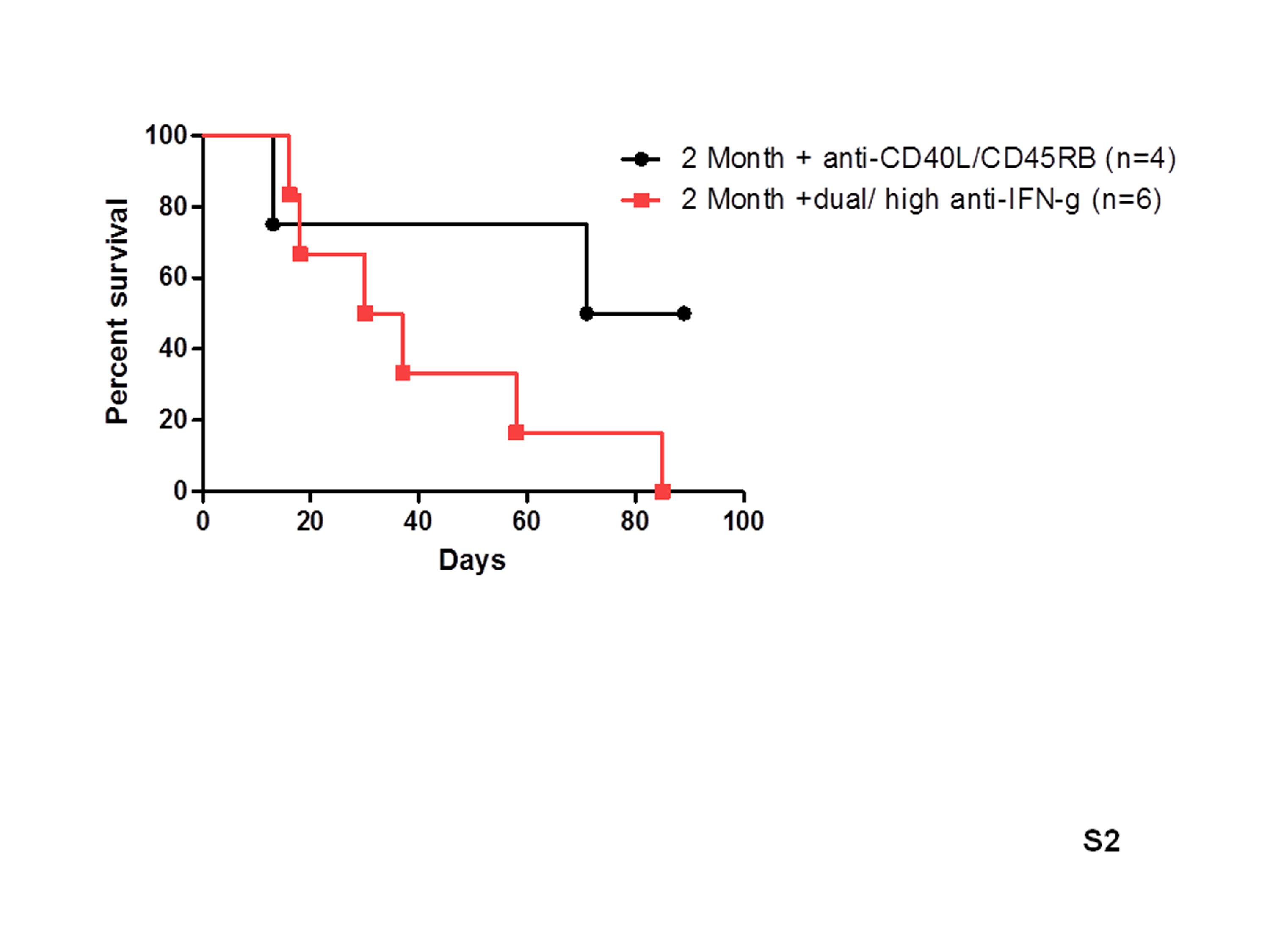

Supplement: Figure S2 — High dose anti-IFN-gamma accelerates graft rejection. Young C57BL/6 mice were grafted with C3H/HeJ skin and treated with anti-CD40L plus anti-CD45RB antibodies with or without anti-IFN-gamma antibody. Recipients receiving additional IFN-gamma antibody exhibited accelerated graft rejection. (TIF) [file pone.0082856.s002.tif]
